# Validity assessment of the PROMIS fatigue domain among people living with HIV

**DOI:** 10.1186/s12981-017-0146-y

**Published:** 2017-04-11

**Authors:** L. E. Gibbons, R. Fredericksen, D. S. Batey, L. Dant, T. C. Edwards, K. H. Mayer, W. C. Mathews, L. S. Morales, M. J. Mugavero, F. M. Yang, E. Paez, M. M. Kitahata, D. L. Patrick, H. M. Crane, P. K. Crane, for the Centers for AIDS Research Network of Integrated Clinical Systems (CNICS)

**Affiliations:** 1 Department of Medicine, Harborview Medical Center, University of Washington, 325 9th Ave, Box 359931, Seattle, WA USA; 2 Department of Social Work, University of Alabama at Birmingham, 900 13th Street S, Suite 203, Birmingham, AL 35294 USA; 3 Fenway Institute, 1340 Boylston Street, Boston, MA 02215 USA; 4 Department of Health Services, University of Washington, 1208 NE 43rd Street, Seattle, WA 98195 USA; 5 Department of Medicine, Harvard Medical School, Boston, MA USA; 6 Department of Medicine, University of California at San Diego, 4168 Front Street, San Diego, CA 92103 USA; 7 Center for Health Equity, Diversity and Inclusion, School of Medicine, University of Washington, 1959 Pacific Street, Seattle, WA 98195 USA; 8 Department of Medicine, University of Alabama at Birmingham, 908 20th Street S, Suite 250, Birmingham, AL 35205 USA; 9 Department of Biostatistics and Epidemiology, Augusta University, 1120 15th Street, AE-1035, Augusta, GA 30912 USA; 10 Harborview Medical Center, University of Washington, 325 9th Ave, Box MS 35978, Seattle, WA 98104 USA

**Keywords:** Fatigue, HIV, PROMIS, Validity, Psychometrics, Measurement, Patient burden

## Abstract

**Purpose:**

To evaluate psychometric characteristics and cross-sectional and longitudinal validity of the 7-item PROMIS^®^ Fatigue Short Form and additional fatigue items among people living with HIV (PLWH) in a nationally distributed network of clinics collecting patient reported data at the time of routine clinical care.

**Methods:**

Cross-sectional and longitudinal fatigue data were collected from September 2012 through April 2013 across clinics participating in the Centers for AIDS Research Network of Integrated Clinical Systems (CNICS). We analyzed data regarding psychometric characteristics including simulated computerized adaptive testing and differential item functioning, and regarding associations with clinical characteristics.

**Results:**

We analyzed data from 1597 PLWH. Fatigue was common in this cohort. Scores from the PROMIS^®^ Fatigue Short Form and from the item bank had acceptable psychometric characteristics and strong evidence for validity, but neither performed better than shorter instruments already integrated in CNICS.

**Conclusions:**

The PROMIS^®^ Fatigue Item Bank is a valid approach to measuring fatigue in clinical care settings among PLWH, but in our analyses did not perform better than instruments associated with less respondent burden.

**Electronic supplementary material:**

The online version of this article (doi:10.1186/s12981-017-0146-y) contains supplementary material, which is available to authorized users.

## Background

Fatigue is a common clinical symptom and adversely impacts health-related quality of life. Fatigue is highly prevalent among persons living with HIV (PLWH) [1, 2]. It is a common side-effect of antiretroviral medications [3], and it is associated with several adverse clinical outcomes, including longer time until depression remission [4], poorer physical functioning [5, 6], poorer adherence to antiretroviral medications [7], and virologic failure [8]. Patients rank fatigue as an important domain for providers to know about in order to provide good care [9].

In many cases fatigue is not systematically assessed as part of clinical care. Challenges that impede fatigue assessment for research in PLWH have been outlined previously, including lack of consistent measurement, lack of longitudinal measurement, and lack of comprehensive clinical data to examine potential predictors of fatigue [10]. Measuring fatigue for clinical care further compounds these issues as there are substantial time constraints and logistical hurdles that must be addressed to minimize impact of assessment on clinical flow.

One option for assessing fatigue among PLWH is the HIV-Related Fatigue Scale [10–12]. This is a well-designed measure with 56 items including subscales addressing concepts such as intensity and impact. Unfortunately, it is too long to be useful in most routine clinical care settings. At the opposite end of the spectrum are very brief assessments such as the single item included in the HIV symptoms index [13].

The Patient Reported Outcomes Measurement and Information System (PROMIS^®^, http://www.nihpromis.org) is a National Institutes of Health Roadmap initiative to develop item banks to measure patient-reported symptoms. PROMIS investigators developed a fatigue item bank [14]. Items from the bank can be used as either a fixed-length short-form or as a computerized adaptive test (CAT) [15]. The PROMIS^®^ Fatigue Item Bank was developed for people in general rather than specific patient groups such as PLWH, which facilitates comparisons with the general population and across patient groups [16]. Well-developed and calibrated universal fatigue measures could enhance comparability of findings and serve as a common metric of fatigue across conditions [15]. Yet, previous analyses of the PROMIS^®^ fatigue domain were not conducted among PLWH, and were not carried out in the context of routine clinical care. We conducted this study to better understand the properties of the PROMIS^®^ fatigue instrument as part of routine clinical care for PLWH.

## Methods

### Study cohort

This study was conducted in the Centers for AIDS Research Network of Integrated Clinical Systems (CNICS) cohort [17], which integrates comprehensive inpatient and outpatient clinical data on PLWH in the cohort [17]. PLWH complete the CNICS clinical assessment of patient reported measures, symptoms, and outcomes (PROs) every 4–6 months as part of routine clinic visits [18, 19]. They use touch screen tablets or personal computers using web-based survey software developed specifically for PROs [18, 20] to complete the clinical assessment which includes a variety of measures such as the HIV Symptoms Index [13], the Patient Health Questionnaire (PHQ-9) [21, 22] for depression, and the modified Alcohol, Smoking, and Substance Involvement Screening Test [23, 24] for illicit drug use. The assessment was integrated into clinical care for regularly scheduled clinic visits at each site. No exclusions were made on the basis of severe fatigue.

### Study participants

PLWH 18 years old or older who spoke English or Spanish at four clinics (University of Washington Madison HIV Clinic, Seattle; University of Alabama at Birmingham 1917 Clinic, Birmingham; University of California San Diego HIV Clinic, San Diego; and Fenway Health, Boston) were eligible to participate in this study. Data were collected from 1597 PLWH from September 2012 to April 2013.

### Qualitative analyses

We conducted in-depth interviews in English and Spanish with 42 patients endorsing fatigue to elicit concepts regarding the experience of living with fatigue and HIV, as described elsewhere [25]. We excerpted and coded transcribed interview content using codes adapted from PROMIS^®^ Fatigue Item Bank content. We matched coded interview content to bank items. The team assessed unmatched content for possible new item development. We reviewed all proposed items using PROMIS^®^ Qualitative Item Review criteria [26], for readability using the Lexile Analyzer, and for translatability into English or Spanish. We held focus groups with 68 patients and asked them to rank-order the prospective item list in order of importance for their provider to know. We retained the most important items and conducted cognitive interviews with 21 patients to assess item comprehensibility, modifying items as needed [25]. We developed four new items in addition to those already in the PROMIS^®^ Fatigue Item Bank [25].

### Item administration

We administered the 7-item PROMIS^®^ Fatigue Short Form [27], an additional 13 items selected from the PROMIS^®^ Fatigue Item Bank (including four items excluded from the final bank and, thus, without PROMIS^®^ item parameters), and our four new items (see Table 1). We modified response options for five existing PROMIS^®^ items because of qualitative feedback. We used PROMIS^®^ item parameters for all of the other PROMIS^®^ items but calibrated the five items with new response options anew.

**Table 1:** Fatigue items administered, with a priori subdomains

Item description	Subdomain
PROMIS 7-item Fatigue Short Form	
How often did you feel tired?	Experience
How often did you run out of energy?	Experience
How often were you too tired to take a bath or shower?	Impact
How often did you experience extreme exhaustion?	Experience
How often did your fatigue limit you at work (including work at home)?	Impact
How often were you too tired to think clearly?	Impact
How often did you have enough energy to exercise strenuously?	Impact
Other calibrated PROMIS items	
How often were you physically drained?	Experience
To what degree did you have to force yourself to get up and do things because of your fatigue?	Impact
How run-down did you feel on average?	Experience
How fatigued were you on average?	Experience
Items calibrated in PROMIS but administered with different response options^a^	
How fatigued were you when your fatigue was at its worst?	Experience
To what degree did your fatigue interfere with your physical functioning?	Impact
I felt fatigued	Experience
I had trouble starting things because I was tired	Impact
How much were you bothered by your fatigue on average?	Experience
Uncalibrated PROMIS items	
How often did you wake up feeling exhausted?	Experience
How often did you feel so exhausted that you stayed in bed all day?	Impact
How often were you too exhausted to take your medication?	Impact
How often were you so exhausted that you missed appointments?	Impact
New items from qualitative interviews	
How often were you too exhausted to carry out your daily responsibilities?	Impact
How often did your body feel exhausted?	Experience
How often were you too exhausted to chew and swallow food?	Impact
How often were you too exhausted to concentrate?	Impact

^a^Because different response options were offered in this study, we did not use the PROMIS item parameters for these items

### Quantitative analyses

We used Stata [28] for all analyses unless otherwise noted.

### Dimensionality

We used structural equation modeling to determine whether the items were sufficiently unidimensional to use item response theory (IRT) in our sample. All structural equation models were fit in Mplus [29]. We applied the following thresholds for acceptable model fit: confirmatory fit index (CFI) > 0.95, Tucker–Lewis index (TLI) > 0.95, and root mean squared error of approximation (RMSEA) < 0.08 [30].

### PROMIS^®^ item parameters

We performed additional analyses to determine whether it was appropriate to use PROMIS^®^ item parameters in our population of PLWH. We initially fixed all seven items from the fatigue short form to their PROMIS^®^ values and used modification indices to identify the item for which constraining parameters to PROMIS^®^ values had the greatest impact on model fit. We then removed those constraints and freely estimated parameters for that item and identified the next item that had the greatest impact on model fit. We repeated this procedure until we were left with two anchor items. We extracted factor scores from the PROMIS^®^-fixed model and from a model with the final two anchor items and five freely estimated items and calculated correlations between these scores. We plotted agreement between scores using a variant of a Bland–Altman plot, with the difference between the scores on the y-axis and the PROMIS^®^-fixed model scores on the x-axis. We superimposed the standard error of measurement (SEM) curve on this graph and examined whether the differences were smaller than the SEM at each level of fatigue.

### Comparison of measurement properties of scores

We computed an IRT score for all 24 items. We fixed item parameters for the 11 PROMIS^®^ items with PROMIS^®^ response options to their PROMIS^®^ values, so scores are on the PROMIS^®^ metric. We freely estimated parameters for the other 13 items. We compared the SEM for the PROMIS^®^-7a short form to that from all 24 items.

### Simulated CAT

We used Firestar [31] to simulate CAT from the 24-item bank we administered. We categorized PLWH into groups based on PROMIS^®^ fatigue scores: <40, 40–50, >50–60, and >60. We set the minimum number of items administered by the simulated CAT at 7 and the default stopping rule of SEM < 0.3 (equivalent: T-metric SEM < 3). We determined the proportion of times each item was administered to people in each fatigue level group. We used seven items as a minimum to determine the extent of overlap between items selected by CAT and items included in the 7-item PROMIS^®^ Fatigue Short Form. As a sensitivity analysis, we performed a second CAT simulation with no minimum number of items and used a 0.3 SEM or seven items maximum stopping rule. We compared patient burden based on the average completion times per item for patients from one site (University of Washington) who completed both instruments, based on the 7-item short form and the number of items in the 2 CAT simulations.

### Differential item functioning (DIF)

We used the Stata command—difwithpar—[32] to evaluate items for DIF with respect to age, sex, race, and nadir CD4 count. We used a *P* value criterion of 0.05 for uniform and for non-uniform DIF. The—difwithpar—algorithm uses demographic-specific item parameters for items identified with DIF and generates new scores that account for DIF. We evaluated DIF impact by comparing naïve scores that ignored DIF to those that accounted for DIF. We use differences of score larger than 0.3 points on the theta metric (larger than 3 points on the T metric) as the primary threshold to indicate salient DIF impact and the median SEM as a more stringent threshold.

### Associations with clinical characteristics

We used Spearman correlations to compare cross-sectional associations between clinical characteristics and the HIV Symptoms Index fatigue item [13], the “tired” item from the PHQ-9 [21, 22], the PROMIS^®^-7a score, and the score derived from the entire 24 items we administered. The clinical characteristics included: hepatitis C virus co-infection; nadir and current CD4 count; the number of symptoms endorsed on the HIV Symptoms Index; specific symptoms endorsed on the HIV Symptoms Index; quality of life estimated using EQ-5D responses [33–36]; and the total PHQ-9 score. Among PLWH taking antiretroviral medications for HIV, we also determined associations between fatigue scores and medication adherence based on the last time the person stated they had missed medications, their self-reported ability to take medications, and the proportion of medications they were estimated to have taken [37–42].

### Test–retest reliability

We had 51 people return to clinic on a second occasion from 6–14 days following their initial assessment to repeat the assessment. Since this involved an extra visit outside the context of clinical care, we provided an incentive of $15 for this activity. We used intraclass correlation coefficients (ICCs) to measure test–retest reliability.

### Longitudinal evaluation

A subset of 249 PLWH had repeat assessment on a second routine clinical care visit from 79–203 days following their initial assessment [median 119 days, interquartile range (IQR) 105–134 days]. Given the episodic nature of HIV symptoms [43], we were interested first in describing changes in fatigue. We also sought to compare changes in fatigue measures in two situations where change might be expected: concurrent with a change in depression symptoms or a change in methamphetamine use on the clinical assessment.

## Results

### Demographic and clinical characteristics from the cross-sectional quantitative data

English questionnaires were completed by 1597 PLWH (Table 2); we included Spanish speakers in our qualitative analyses, but there were too few respondents in Spanish (n = 94) for meaningful quantitative analyses. Mean age (SD) was 45.7 (10.4), with a range from 20 to 83 (IQR 39, 53).

**Table 2:** Participant characteristics (n = 1597)

Characteristic	N	Percent	Recent CD4 <500		Recent CD4 500+	
			N	Percent	N	Percent
Male	1315	82.3	591	84.8	724	80.4
Race						
White	861	53.9	346	49.6	515	57.2
Black	461	28.9	197	28.3	264	29.3
Hispanic	195	12.2	105	15.1	90	10.0
Other	80	5.0	49	7.0	31	3.4
HIV Symptom Index fatigue item						
No fatigue	558	34.9	233	33.4	325	36.1
Doesn’t bother me	151	9.5	66	9.5	85	9.4
Bothers me a little	383	24.0	161	23.1	222	24.7
Bothers me	260	16.3	117	16.8	143	15.9
Bothers me a lot	221	13.8	110	15.8	111	12.3
Did not answer	24	1.5	10	1.4	14	1.6
CD4 Nadir						
<200	732	45.8	487	69.9	245	27.2
200 to <350	392	24.6	152	21.8	240	26.7
350 or higher	468	29.3	55	7.9	413	45.9
Missing	5	0.3	3	0.4	2	0.2
Taking anti-HIV meds	1412	88.4	604	86.7	808	89.8

**Fig. 1:**
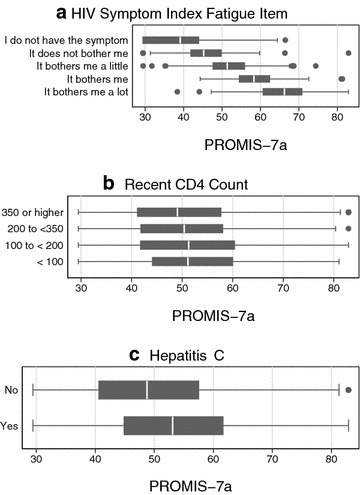
*Box* and* whisker plots* showing the distribution of PROMIS^®^ 7a fatigue scores on the PROMIS^®^ T score metric for each level of fatigue according to the HIV symptoms inventory fatigue item* (**a**); for people with different recent CD4+ T-cell counts (**b**), and with and without Hepatitis C virus co-infection (**c**). *For these plots, the box shows the 25th and 75th percentile scores, and the median is shown with a *white vertical bar* within the *box*. The *whiskers* show 1.5 times the extent of the *box*. *Dots* show more extreme values. In **a**, the median score for the group who denied having fatigue was around 40; the median for those who had fatigue but stated it did not bother them was around 45; the median score for those who had fatigue that bothered them a little was around 53; the median score for those who had fatigue that bothered them was around 58; and the median score for those who had fatigue that bothered them a lot was around 66

Fatigue was common in this cohort. Using the HIV Symptoms Index single item, 65% stated they had fatigue (Table 2). Scores from the PROMIS^®^ items mapped closely to these scores from the HIV Symptoms Index. As shown in Fig. 1, median fatigue scores on the PROMIS^®^ metric ranged from just below 40 for those who stated they did not have fatigue to just over 65 for those who stated they had fatigue and that it bothered them a lot.

### Dimensionality

A single factor confirmatory factor analysis model did not fit well by RMSEA criteria (CFI 0.98, TLI 0.98, RMSEA 0.103). We assigned items a priori to one of two subdomains, the experience of fatigue vs. the impact of fatigue, based on PROMIS^®^’s domain framework (see Table 1), but this model did not fit well and had loadings that did not support the theoretical structure, such as negative loadings on a subdomain. A negative loading means that as levels of the item were of increasing severity, the level of fatigue impact was expected to be *lower down*, which is difficult to explain.

We then considered modification indices from a single factor model that suggested candidate pairs of items with residual correlations that would have the greatest impact on model fit. We included 6 such pairs, which resulted in a model with CFI 0.99, TLI 0.99, and RMSEA 0.08. We extracted factor scores for the single factor score and the bifactor score with the six residual correlations. These scores were highly correlated at 0.9999. We compared standardized factor loadings between these models, and the largest difference was 0.020, well lower than the 0.10 threshold that would indicate a salient difference in loadings between the single factor and bifactor models [44]. These findings led us to conclude that the items were sufficiently unidimensional to proceed with IRT analyses.

### PROMIS item parameters

The loadings and thresholds for the two anchor items and the five freely estimated items are shown in Additional file 1. The correlation between the score using those parameters and the score based entirely on PROMIS parameters was >0.99. All of the score differences were within the SEM curve thresholds (Additional file 2). These results supported use of PROMIS item parameters for PLWH.

### Measurement properties

**Fig. 2:**
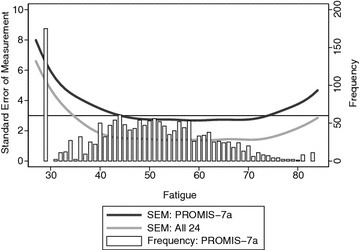
Histogram of observed fatigue levels (*open bars*) superimposed on standard error of measurement curves for the 24 fatigue items administered to participants (*lower light gray curve*) and for the 7-item PROMIS^®^ Fatigue Short Form (*upper darker gray curve*). A *horizontal line* is at a SEM of 3, which is the common default stopping rule for computerized adaptive testing

We show a plot of the SEM for the 24 items administered and for the 7-item PROMIS^®^ Fatigue Short Form subset in Fig. 2. The median SEM was 0.29 (range 0.24–0.57; IQR 0.26–0.34) for the 7-item PROMIS^®^ Fatigue Short Form and 0.15 (range 0.11–0.52; IQR 0.14–0.20) using all 24 items. On the T-score metric, the 7-item PROMIS^®^ Fatigue Short Form has an SEM < 3 over the 45–73 range, while using all 24 items gives an SEM under 3 for all scores 35 and above. We also show a histogram of observed fatigue scores from the 7-item PROMIS^®^ Fatigue Short Form on the same plot. There are very few people with extremely high levels of fatigue (over 73) for whom the 24 items would provide a markedly improved level of precision; most of the people for whom differences in precision between the 7-item short form and the 24-item bank are characterized by low levels of fatigue, with scores 35-45 on the PROMIS^®^ metric. While scores in this range are common, it may not be clinically important to measure fatigue levels precisely in these individuals 1.5–2.5 SD below national norms for fatigue.

### CAT results

Our first CAT simulation used a minimum of seven items. With this criterion, only people with PROMIS fatigue scores <40 required more than seven items to achieve a SEM < 3 on the T-metric (Table 3). There were two items that were administered in all simulated CATs: “*How run*-*down did you feel on average*” and “*How fatigued were you on average*.” The item “*How often were you physically drained*” was almost always administered. None of the items from the 7-item PROMIS^®^ Fatigue Short Form was routinely selected for CAT administration across all fatigue levels, though “*How often did you feel tired*” and “*How often did you run out of energy*” were always administered to individuals with fatigue scores <40.

**Table 3:** Frequency of item administration in simulated computerized adaptive testing, by level of fatigue

Item	Overall (n = 1597)	Fatigue score			
		≤40 (n = 246)	>40–50 (n = 561)	>50–60 (n = 556)	>60 (n = 234)
How often did you feel tired?	22.0	100.0	18.4	0.0	1.3
How often did you run out of energy?	57.5	100.0	54.2	45.9	48.7
How often were you too tired to take a bath or shower?	6.6	43.1	0.0	0.0	0.0
How often did you experience extreme exhaustion?	7.3	45.9	0.0	0.0	1.7
How often did your fatigue limit you at work (including work at home)?	8.9	57.7	0.0	0.0	0.0
How often were you too tired to think clearly?	7.8	45.9	0.0	0.0	4.7
How often did you have enough energy to exercise strenuously?	8.7	56.5	0.0	0.0	0.0
How often were you physically drained?	99.7	100.0	100.0	100.0	97.9
To what degree did you have to force yourself to get up and do things?	79.8	46.7	69.5	100.0	91.5
How run-down did you feel on average?	100.0	100.0	100.0	100.0	100.0
How fatigued were you on average?	100.0	100.0	100.0	100.0	100.0
How fatigued were you when your fatigue was at its worst?	11.6	75.2	0.0	0.0	0.0
To what degree did your fatigue interfere with your physical functioning?	70.6	49.6	38.3	100.0	100.0
I felt fatigued	47.3	64.2	82.7	22.1	4.3
I had trouble starting things because I was tired	20.5	48.0	0.0	35.1	6.0
How much were you bothered by your fatigue on average?	85.2	85.4	75.6	89.9	97.0
How often did you wake up feeling exhausted?	7.6	49.2	0.0	0.0	0.0
How often did you feel so exhausted that you stayed in bed all day?	6.9	44.7	0.0	0.0	0.0
How often were you too exhausted to take your medication?	6.2	40.2	0.0	0.0	0.0
How often were you so exhausted that you missed appointments?	6.6	42.7	0.0	0.0	0.0
How often were you too exhausted to carry out your daily responsibilities?	7.0	43.9	0.0	0.0	1.7
How often did your body feel exhausted?	46.1	100.0	61.3	7.0	46.2
How often were you too exhausted to chew and swallow food?	6.1	39.8	0.0	0.0	0.0
How often were you too exhausted to concentrate?	7.0	45.5	0.0	0.0	0.0
Mean number of items	8.3	15.2	7.0	7.0	7.0
Mean standard error of measurement (T-metric)	2.2	3.2	2.1	2.0	2.1

As outlined in our previous publication, we developed four new fatigue items based on our qualitative work [25]. In simulated CAT, one of these, “*How often did your body feel exhausted?*” was selected 46% of the time; it was always selected for people with fatigue scores ≤40, 61% of the time for fatigue scores >40–50 and 46% of the time for those with the highest levels of fatigue. In contrast, the other new items we developed were either never or rarely selected for people with fatigue levels >40; these items were “*How often were you too exhausted to carry out your daily responsibilities*?”, “*How often were you too exhausted to chew and swallow food?*” and “*How often were you too exhausted to concentrate?*”

In our secondary analyses, we completed another CAT simulation with no minimum number of items and a stopping rule of either a standard error of measurement <3 points on the T metric or up to 7 items maximum; the median (IQR) number of items administered was 3 (3–4).

Based on the mean time per item for the PROMIS fatigue items (mean 6.73 s, SD 2.74 per item), a person completing the 7-item PROMIS short form or 7-item CAT would be expected to take an average of 47.1 s. Based on the second simulated CAT where people completed a mean of 3 items, the average completion time for the PROMIS fatigue CAT would be 20.2 s. This is in comparison to an estimated time to complete the HIV Symptom Index fatigue screening item of 6 s (mean 6.0 s, SD 10.1).

### DIF results

A few items had DIF with respect to age, sex, race, and/or nadir CD4 count with the very sensitive DIF thresholds we used (results not shown). There was negligible DIF impact, and for none of these covariates was there any individual PLWH where accounting for DIF led to a change in score as much as three points on the PROMIS T-score metric. Indeed, when we considered a more stringent 1.7 points PROMIS T-score metric (the median SEM for this sample), only 1–7 people (all <1%) had DIF impact of this magnitude with respect to each of these covariates. We concluded that there was negligible DIF in these items with respect to these covariates.

### Associations with clinical characteristics

The HIV Symptom Index single item fatigue score was closely associated with the 7-item PROMIS^®^ Fatigue Short Form (ρ = 0.82) and with the score from all of 24 items (ρ = 0.85) (Table 4). Similarly, the PHQ-9 fatigue item was closely associated with the HIV Symptoms Index fatigue item (ρ = 0.77), with the 7-item PROMIS^®^ Fatigue Short Form (ρ = 0.75), and with the 24-item score (ρ = 0.77). Correlations with clinical characteristics were generally as strong for the HIV Symptom Index fatigue item as they were for either the 7-item PROMIS^®^ Fatigue Short Form or the full 24-item score.

**Table 4:** Spearman correlation coefficients between fatigue measures and clinical characteristics

	HIVSI fatigue		PHQ-9 “tired”		PROMIS-7a		All 24 items	
	Coefficient	P value	Coefficient	P value	Coefficient	P value	Coefficient	P value
Factors related to fatigue itself								
HIV Symptom Index 1-item Fatigue response	–	–	0.77	<0.0001	0.82	<0.0001	0.85	<0.0001
PHQ-9 item “feeling tired or having little energy”	–	–	–	–	0.75	<0.0001	0.77	<0.0001
Hepatitis-C	0.09	0.0004	0.09	0.0002	0.11	<0.0001	0.12	<0.0001
HIV disease severity								
Current CD4 count	−0.04	0.1402	−0.04	0.1562	−0.05	0.0699	−0.04	0.2392
CD4 nadir	0.01	0.8828	0.05	0.0534	0.00	0.8584	0.01	0.3481
Symptom burden								
Number of symptoms in inventory	0.69	<0.0001	0.59	<0.0001	0.64	<0.0001	0.66	<0.0001
Specific symptoms								
Fever, chills or sweats	0.42	<0.0001	0.37	<0.0001	0.64	<0.0001	0.44	<0.0001
Dizzy or lightheaded	0.51	<0.0001	0.46	<0.0001	0.42	<0.0001	0.54	<0.0001
Pain/numbness/tingling in hands/feet	0.44	<0.0001	0.35	<0.0001	0.52	<0.0001	0.49	<0.0001
Having difficulty remembering	0.57	<0.0001	0.48	<0.0001	0.46	<0.0001	0.62	<0.0001
Nausea or vomiting	0.45	<0.0001	0.37	<0.0001	0.63	<0.0001	0.47	<0.0001
Diarrhea or loose bowel movements	0.41	<0.0001	0.34	<0.0001	0.47	<0.0001	0.42	<0.0001
Feeling sad or depressed	0.65	<0.0001	0.55	<0.0001	0.40	<0.0001	0.66	<0.0001
Feeling nervous or anxious	0.57	<0.0001	0.50	<0.0001	0.63	<0.0001	0.61	<0.0001
Difficulty falling or staying asleep	0.57	<0.0001	0.50	<0.0001	0.58	<0.0001	0.59	<0.0001
Coughing or trouble catching breath	0.39	<0.0001	0.36	<0.0001	0.56	<0.0001	0.44	<0.0001
Headaches	0.42	<0.0001	0.35	<0.0001	0.43	<0.0001	0.46	<0.0001
Appetite loss/change taste of food	0.45	<0.0001	0.43	<0.0001	0.44	<0.0001	0.52	<0.0001
Bloating pains or gas in stomach	0.44	<0.0001	0.37	<0.0001	0.51	<0.0001	0.47	<0.0001
Muscle aches or joint pain	0.47	<0.0001	0.42	<0.0001	0.47	<0.0001	0.52	<0.0001
Sex-loss of interest/satisfaction	0.47	<0.0001	0.41	<0.0001	0.50	<0.0001	0.50	<0.0001
Body changes-fat deposits/wt gain	0.46	<0.0001	0.38	<0.0001	0.50	<0.0001	0.49	<0.0001
Weight loss or wasting	0.32	<0.0001	0.30	<0.0001	0.49	<0.0001	0.39	<0.0001
EQ-5D	0.59	<0.0001	0.56	<0.0001	0.38	<0.0001	0.64	<0.0001
PHQ-9	0.75	<0.0001	0.84	<0.0001	0.63	<0.0001	0.77	<0.0001
Self-rated health (EuroQOL)	−0.53	<0.0001	0.50	<0.0001	0.74	<0.0001	−0.57	<0.0001
Among ART users								
Last time missed meds	−0.15	<0.0001	−0.16	<0.0001	−0.18	<0.0001	−0.19	<0.0001
Ability to take meds	−0.19	<0.0001	−0.24	<0.0001	−0.23	<0.0001	−0.23	<0.0001
Take how much of meds	−0.14	0.0001	−0.16	<0.0001	−0.17	<0.0001	−0.17	<0.0001

### Test–retest reliability

Fifty-one people completed the 7-item PROMIS^®^ Fatigue Short Form again 6–14 days later (median 8, IQR 7–11 days). The ICC was 0.74 (0.55, 0.83). The mean change was −0.17 points, though 4 people had a decrease of at least on point and 2 had an increase of at least one point, either due to true changes in fatigue [43] or measurement error. Among the 31 people who said their level of fatigue was “about the same” as previously, the ICC was similar at 0.66 (0.44, 0.81).

### Longitudinal analyses

On average there was little change in level of fatigue over approximately 4 months—the mean change was −0.16. However, this obscures individual variation, in that 9% reported an increase in fatigue of at least one point, and 16% reported a decrease of at least one point. Changes in the PHQ-9 depression score were more highly correlated with changes in the HIV Symptom Index fatigue item (Spearman ρ = 0.47) than were changes in the 7-item PROMIS^®^ Fatigue Short Form score (ρ = 0.39). Only 13 people changed from using methamphetamines to not, or vice versa, so comparisons of the fatigue measures were not feasible.

## Discussion

In a thorough evaluation of the psychometric properties of the 7-item PROMIS^®^ Fatigue Short Form and additional items selected from the PROMIS^®^ Fatigue Item Bank or items specifically developed for this project, we found that these fatigue items had excellent content validity among PLWH. While the 24 fatigue items did not form a scale that was strictly unidimensional, it was sufficiently unidimensional to use item response theory. Furthermore, our analyses suggested that PROMIS^®^ item parameters were appropriate to use among PLWH. We used very sensitive DIF detection thresholds and identified items with DIF, but did not find salient impact for DIF with respect to age, sex, race, or nadir CD4 count. Scores from the 7-item PROMIS^®^ Fatigue Short Form or from all 24 items from the fatigue item bank had excellent validity in a variety of analyses, but were no better than the HIV Symptom Index single fatigue item measure or the fatigue item from the PHQ-9. The HIV Symptom Index single fatigue item has limited ability to detect change over time, because it has only a few response options. Nevertheless, in the longitudinal sample, we did not find evidence that the PROMIS scores were more responsive to change than was the HIV Symptom Index fatigue item or the PHQ-9 fatigue item.

Fatigue is clearly a relevant consideration for this clinical population. Sizable numbers of PLWH had substantial levels of fatigue. One advantage of the PROMIS^®^ fatigue metric is that we can relate fatigue levels to national averages. As shown in Fig. 1 and in Table 3, substantial numbers of PLWH endorse high levels of fatigue. Those who stated that they have fatigue that bothers them a lot on the HIV Symptom Index have median (IQR) PROMIS^®^ fatigue scores of 66 (IQR 61–71), which is about 1.5 SD (1–2 SD) above the national average.

Our CAT simulations showed a small amount of overlap with the 7-item PROMIS^®^ Fatigue Short Form. We set up the first simulation such that each individual received at least seven items to facilitate comparisons with the short form. Only people with very low fatigue levels received more than 7 items from the simulated CAT; everyone else received exactly 7 items. While the 7-item short form may not include the most informative items from the PROMIS^®^ Fatigue Item Bank, it nevertheless had good measurement precision across a broad range of fatigue levels (see Fig. 2). Furthermore, the 7-item PROMIS^®^ Fatigue Short Form performed well in all of our validity analyses; indeed, scores from the 7-item PROMIS^®^ Fatigue Short Form performed just as well as scores from the entire 24 items we considered. At the same time, briefer instruments, including the fatigue item from the PHQ-9 and the single HIV Symptom Index fatigue item, also did well in all of our validity analyses. We did not find a compelling case to choose the PROMIS^®^ fatigue scores over much shorter instruments. A CAT with different specifications could have arrived at a PROMIS fatigue score in fewer items, but it would be unlikely to have better performance in our validity analyses than the entire scale considered here. Furthermore, the HIV Symptom Index fatigue item required much less time on average for patients to complete than the 7-item PROMIS short form, CAT, or even the shorter CAT with an average of 3 items. While this may be of limited importance in research settings, minimizing patient burden in clinical care settings is important to avoid impacting clinical flow.

Our findings should be considered in the context of strengths and limitations. Our study was performed in CNICS, which is a nationally distributed cohort of PLWH who are in clinical care. Our data were collected from convenience samples of PLWH seen in particular calendar months, and were not purposefully sampled from people particularly likely to have changing fatigue levels. Generalizability is limited as our study was conducted only among PLWH. We did administer the PROMIS fatigue items to Spanish speakers, but had too few of them during the data collection window to facilitate analyses of DIF. We found no evidence of DIF with respect to four covariates, but were not able to evaluate DIF with respect to Spanish vs. English. The CNICS assessment of patient-reported measures now includes Amharic, but unfortunately, an Amharic version of the PROMIS Fatigue Item Bank has not been developed, nor were we able to assess the performance of these items in any other language.

Our ability to evaluate change in fatigue over time was limited, because we had few options for external comparison. One validation option was changes in depression levels as measured by the PHQ-9, where we found that the HIV Symptom Index fatigue item was more closely correlated to changes in depression levels than were PROMIS scores. In theory, IRT scores are more accurate measures of change over time than ordinal scales, because they have linear measurement properties [45], which means that one point of change in a score corresponds to the same amount of change in fatigue regardless of the initial level of fatigue. Indeed, PROMIS^®^ scores may have shown better responsiveness to change than the HIV symptoms index fatigue item scores had we designed our study specifically to collect data on people expected to change [46]. In that setting, a brief CAT may prove to have better responsiveness to change than the single HIV symptoms index fatigue item and may fit in a reasonable time footprint, making this a feasible choice in routine clinical care settings. Firmer conclusions regarding responsiveness of PROMIS^®^ scores among PLWH will require additional data.

This study has several strengths that are also worth noting. It includes a particularly relevant population (PLWH) given the high rates of fatigue experienced by a substantial proportion of this group. We studied the performance of these items in a geographically and racially/ethnically diverse population. We performed a variety of psychometric analyses using state-of-the-art approaches.

Fatigue in PLWH often does not remit [10], suggesting the need for additional research to better understand factors leading to fatigue in PLWH and interventions to successfully address it. Research on fatigue among PLWH will require a sustainable systematic approach to measuring fatigue in clinical care.

## Conclusions

The PROMIS^®^ Fatigue Short Form and other fatigue items performed well among PLWH, though we did not find evidence that they performed better than shorter legacy scales in the specific context of routine clinical care. Unless comparison to national norms is needed, the HIV Symptom Index fatigue item may be preferred in HIV clinical care settings due to reduced patient burden.

## Additional files

**Additional file 1.** Loadings and thresholds for Mplus based on the PROMIS item bank parameters, and using 2 anchor items and 5 freely estimated items.

**Additional file 2.** Difference in scores between the PROMIS-7a scored using PROMIS item parameters and a score where 2 items are fixed to the PROMIS item parameters and the other 5 are freely estimated. The horizontal line at zero represents no difference, and the upper and lower curves represent the standard error of measurement. All differences are within the standard error of measurement curves.

## Abbreviations

CAT: computerized adaptive test
CFI: confirmatory fit index
CNICS: Centers for AIDS Research Network of Integrated Clinical Systems
DIF: differential item functioning
ICC: intraclass correlation coefficient
IQR: interquartile Range
IRT: item response theory
PHQ-9: patient health questionnaire for depression
PLWH: persons living with HIV
PROMIS: Patient Reported Outcomes Measurement and Information System
PROs: patient reported measures, symptoms, and outcomes
RMSEA: root mean squared error of approximation
SEM: standard error of measurement
TLI: Tucker–Lewis Index
