# Supplementary material for: Validity assessment of the PROMIS fatigue domain among people living with HIV
Source: AIDS Res Ther. 2017 Apr 11;14:21. doi: 10.1186/s12981-017-0146-y (PMC5387298; doi:10.1186/s12981-017-0146-y)
Supplement: Supplementary file 1 — Additional file 1. Loadings and thresholds for Mplus based on the PROMIS item bank parameters, and using 2 anchor items and 5 freely estimated items. [file 12981_2017_146_MOESM1_ESM.pdf]

Validity assessment of the PROMIS Fatigue domain among people living with HIV

LE Gibbons, R Fredericksen, DS Batey, L Dant, TC Edwards, KH Mayer, WC Mathews, LS Morales, MJ Mugavero, FM Yang, E Paez, MM Kitahata, DL Patrick, HM Crane, PK Crane

Corresponding Author: Laura Gibbons, gibbonsl@u.washington.edu

Additional file 1.

Loadings and thresholds for Mplus based on the PROMIS item bank parameters, and using 2 anchor items and 5 freely estimated items.

	PROMIS item bank parameters					Only 2 items fixed				
	Loading	Thresholds				Loading	Thresholds			
How often did you feel tired?	1.000	-0.978	-0.395	0.445	1.252	.956	-1.549	-0.457	0.697	1.712
How often did you run out of energy?	1.035	-0.764	-0.145	0.612	1.391	.959	-0.972	0.037	1.036	2.074
How often were you too tired to take a bath or shower?	0.884	0.238	0.722	1.293	1.876	.904	0.346	1.026	1.881	2.995
How often did you experience extreme exhaustion?	0.960	0.100	0.635	1.174	1.857	.936	-0.105	0.785	1.608	2.654
How often did your fatigue limit you at work (including work at home)?	0.951	-0.517	0.310	1.289	2.211	.951	-0.517	0.310	1.289	2.211
How often were you too tired to think clearly?	0.948	-0.101	0.776	1.721	2.891	.948	-0.101	0.776	1.721	2.891
How often did you have enough energy to exercise strenuously?	0.276	-0.392	0.208	0.871	1.646	.761	-1.890	-0.640	0.256	1.136

The fifth and sixth items (in red), "*How often did your fatigue limit you at work (including work at home)?*" and "*How often were you too tired to think clearly?*" were the anchor items. In PROMIS, "*How often did you have enough energy to exercise strenuously?*" had a much weaker loading than the other items, and in our data the item was weighted even less.
