# Supplementary material for: Validity assessment of the PROMIS fatigue domain among people living with HIV
Source: AIDS Res Ther. 2017 Apr 11;14:21. doi: 10.1186/s12981-017-0146-y (PMC5387298; doi:10.1186/s12981-017-0146-y)

Validity assessment of the PROMIS Fatigue domain among people living with HIV

LE Gibbons, R Fredericksen, DS Batey, L Dant, TC Edwards, KH Mayer, WC Mathews, LS Morales, MJ Mugavero, FM Yang, E Paez, MM Kitahata, DL Patrick, HM Crane, PK Crane

Corresponding Author: Laura Gibbons, gibbonsl@u.washington.edu

Additional file 2.

Figure. Difference in scores between the PROMIS-7a scored using PROMIS item parameters and a score where 2 items are fixed to the PROMIS item parameters and the other 5 are freely estimated. The horizontal line at zero represents no difference, and the upper and lower curves represent the standard error of measurement. All differences are within the standard error of measurement curves.

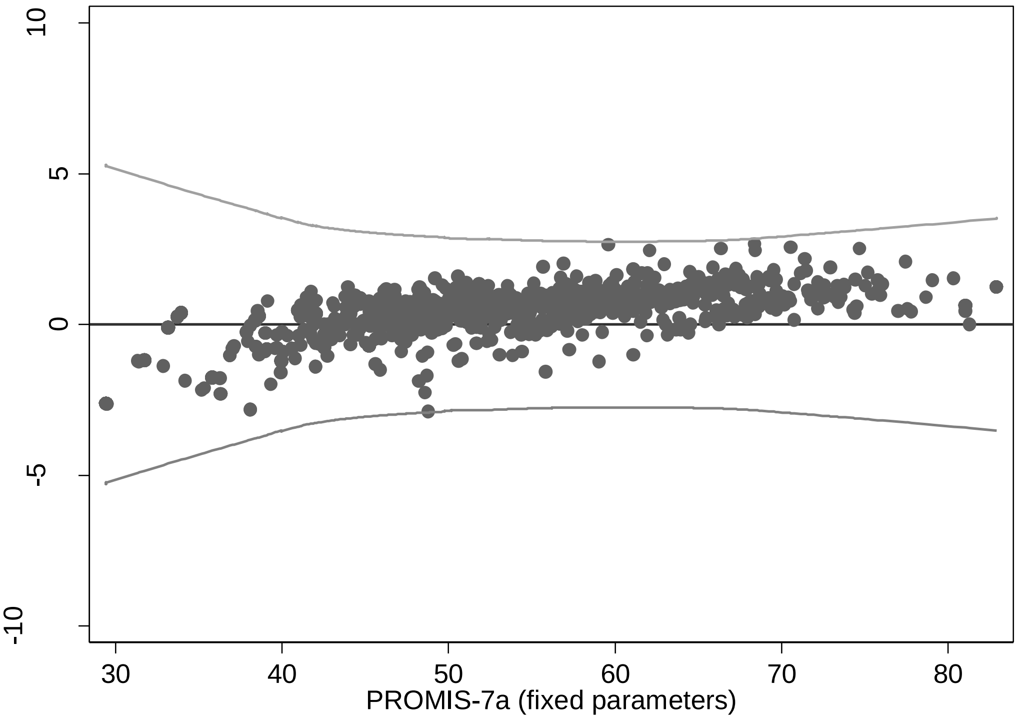

Supplement: Supplementary file 2 — Additional file 2. Difference in scores between the PROMIS-7a scored using PROMIS item parameters and a score where 2 items are fixed to the PROMIS item parameters and the other 5 are freely estimated. The horizontal line at zero represents no difference, and the upper and lower curves represent the standard error of measurement. All differences are within the standard error of measurement curves. [file 12981_2017_146_MOESM2_ESM.pdf]
